# Artificial Neural Network in Fibres Length Prediction for High Precision Control of Cellulose Refining

**DOI:** 10.3390/ma12223730

**Published:** 2019-11-12

**Authors:** Daniele Almonti, Gabriele Baiocco, Vincenzo Tagliaferri, Nadia Ucciardello

**Affiliations:** 1 Department of Enterprise Engineering “Mario Lucertini”, University “Tor Vergata”, Via del Politecnico 1, 00133 Roma, Italy; daniele.almonti@uniroma2.it (D.A.); tagliaferri@mec.uniroma2.it (V.T.); 2 Department of Engineering, University “Roma Tre”, Via Vito Volterra 62, 00146 Roma, Italy; gabriele.baiocco@unioma2.it

**Keywords:** artificial neural networks, machine learning, cellulose fibres processing, process management, refining optimisation

## Abstract

Paper, a web of interconnected cellulose fibres, is widely used as a base substrate. It has been applied in several applications since it features interesting properties, such as renewability, biodegradability, recyclability, affordability and mechanical flexibility. Furthermore, it offers a broad possibility to modify its surface properties toward specifics additives. The fillers retention and the fibres bonding ability are heavily affected by the cellulose refining process that influences chemical and morphological features of the fibres. Several refining theories were developed in order to determine the best refining conditions. However, it is not trivial to control the cellulose refining as different phenomena occur simultaneously. Therefore, it is intuitively managed by experienced papermakers to improve paper structures and properties. An approach based on the machine learning aimed at estimating the effects of refining on the fibres morphology is proposed in this study. In particular, an artificial neural network (ANN) was implemented and trained with experimental data to predict the fibres length as a function of refining process variables. The prediction of this parameter is crucial to obtain a high-performance process in terms of effectiveness and the optimisation of the final product performance as a function of the process parameter. To achieve these results, data mining of the experimental patterns collected was exploited. It led to the achievement of excellent performance and high accuracy in fibres length prediction.

## 1. Introduction

In its essence, paper is a network of fibres interconnected. Usually, it is filled with several additives aimed at controlling the penetration of coating colours and ink, as well as improving mechanical performance and morphological features. In order to ensure effective additives action and efficient manufacturing processes, a high retention ability of a fibres web is required. Furthermore, for reaching elevated mechanical performance, fibres must feature strong linkages. These paper properties can be deeply affected by the manufacturing process, as it influences the fibres specification, such as morphology and surface chemistry.

The raw materials exploited for the paper production include softwoods, hardwoods and nonwood fibres. The main elements of the fibres may be identified in cellulose, hemicellulose and lignin [1]. Fibres are structured in elementary fibrils, which are twisted around the cell wall axis. The structural formula of microfibrils presents hydroxyl groups in the glucose units, which are responsible for the hydrogen bonding ability and allow for the creation of a strong paper structure [2,3,4]. Further, since ionisable acidic groups are present in lignin and hemicellulose, when fibres are suspended in water, they carry a negative charge. The fibres charge and the electrochemical parameters of a pulp are fundamental in papermaking, as a large number of the interactions between fibres and fillers are charge-induced.

Cellulose refining is a critical step in paper production. During the refining, compression and shear forces are applied to fibres flocs, causing several changes in fibres specifications, such as fibres morphology and distribution of chemicals on the surface of the fibres. Fibres morphological changes have been widely discussed. Particularly, they can be summarised in external and internal fibrillation and fines formation, in addition to fibres shortening and straightening [1]. Internal fibrillation is the effect of delamination of the outer layers of the fibres as a consequence of the cyclic compression action of the forces inside the refining devices [5]. The breakage of the bonds among the three constitutive elements of the fibres causes expansion and swelling of the porous structure inside the cell wall, making the fibres more flexible. In addition, studies demonstrated that layers delaminated during the refining process are major restrains against swelling [6,7]. The leakage of fibrils from the surface of the fibres is defined as external fibrillation, and its main result is the growth of the specific surface area. It is always associated with internal fibrillation and fines formation, since they occur simultaneously. Therefore, it is hard to judge the external fibrillation role within the refining process [8]. Further, growing amounts of charged fines are produced. In [9], fines are defined as the fraction of a pulp that is able to pass through a mesh screen or a perforated plate having a hole diameter of 76 µm. The secondary fines formation is the effect of external fibrillation or fibres shortening during the refining process. They are different from the primary fines, which come from ray cells and parenchyma cells, and are present also in nonrefined pulps. The primary and secondary fines are usually characterised by a reduced length, generally less than 0.3 mm, and their presence within the pulp causes a greater bonding capacity as well as a reduction in drainage speed [9,10,11]. The last effect of refining is the fibres length modification as a consequence of the shear and compression forces applied. Fibres lengthening is critical for producing high-quality products, since it results in an enhanced load capacity, stress distribution along the network and elastic modulus [12]. Excessive fibres lengthening or high shear forces may result in fibres shortening. Although the latter is often considered as a detrimental effect, in some rare applications, it may be desirable [13]. As the fibres shortening is related to fines formation, it is difficult to measure accurately the length changes after refining treatment. Usually, length changes are evaluated considering the weighted average lengths before and after refining [14]. In addition, morphological changes of the fibres affect the chemicals and charge distribution on fibres surfaces and walls. Indeed, the morphological changes of fibres make the chemical constituents of the fibres more accessible. However, a relationship between chemical changes and variation of the fibre surface charge during refining has not been established yet [15]. 

From the above review, it is clear that, in the context of industrial paper production, fibres refining represents one of the greatest challenges in order to obtain high-quality products. Overall, all the aforementioned effects of refining occur simultaneously, influencing each other. It is evident that the refining effects on fibres charge and morphology are extremely complex, since both chemical and mechanical factors are involved. Additionally, they are correlated by means of a nonlinear relationship. Several refining theories were developed in order to determine the most suitable refining control system and the best refining conditions. The most commons are based on the energy applied to the fibres during the process. Therefore, it is the most exploited variable to define the refining intensity [16,17,18]. These theories also consider parameters such fibre lengths, coarseness, refining disks geometry and rotation speeds, in addition to pulp consistency. However, other theories consider the forces in order to characterise the refining action [19,20,21].

Although these several refining theories and methods to explore how a three-dimensional paper structure is related to macroscopic paper properties are now emerging, alternative indirect methods were applied over years. However, no theories concerning the morphological modification of refined fibres were reported. Therefore, the manufacturing process is often managed by experienced papermakers that know intuitively how to vary the refining settings to improve the paper properties. The knowledge is important, despite being more empirical than scientific. In the context of artificial intelligence (AI), expert systems and artificial neural networks (ANNs) have been widely employed to exploit the empirical knowledge in the management of industrial production systems. The ANNs have been widely applied over years; particularly, they were applied in modelling, pattern classification and clustering tasks [22]. There are several application fields, including aid of decision-making processes [23,24,25,26], classification tasks [27,28,29], phenomena and processes prediction [30,31,32,33,34], design optimisation [35,36] and materials characterisation [37,38].

Several works in the literature investigate an ANN approach to paper-making. These methods mainly concern the application of neural models for paper properties prediction [39,40,41,42,43]; others deal with pulping process control [44,45]. To the best of our knowledge, no work concerning the prediction of fibres morphology, and specifically fibres lengths, by means of AI has been presented.

The objective of the proposed work is the implementation of a machine learning system addressed towards the control of the refining process. Particularly, the implementation of an ANN aimed at predicting the length of the outgoing fibres from a refining device is presented. The possibility of foreseeing one of the fundamental morphological characteristics of the fibres would be crucial for optimising the product performance and manufacturing the process efficiency. In this respect, the collection and the analysis of an experimental dataset allowed for the training of the ANN implemented. The result achieved showed an excellent ability in the prediction of the fibres length after a refining process on the basis of the main process parameters involved in the paper production. Therefore, the application of ANNs is a valuable tool for the innovation of the traditional control methods aimed at optimising the manufacturing process and the final products performance. 

## 2. Materials and Methods

**Figure 1:**
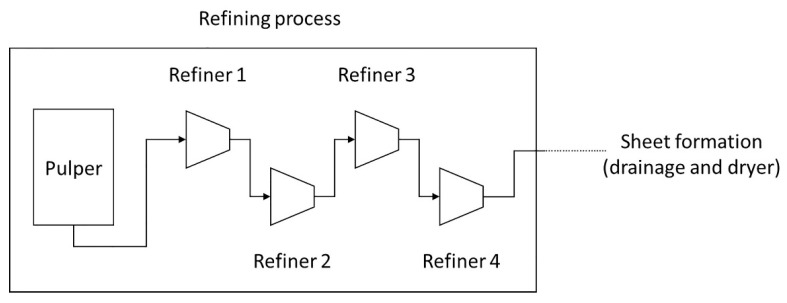
Schematic of a refining process.

The refining analysis was carried out considering a system composed of four conical refiners, as represented in Figure 1. 

Outgoing pulp samples from refiners and outgoing untreated pulp from a pulper were collected and analysed. Initially, the main variables affecting the refining process were identified. In particular, three typologies of variables were defined. The first group of variables concerned the pulp composition in terms of fibres content and fillers amount, which remained constant for all the refining phases. The fibres were classified both qualitatively and quantitatively, reporting their amounts as a percentage of the total quantity of the pulp. The fibres qualities exploited in the paper production during analysis performed were as follows: Pacifico, FSC Cenibra, A3F from Aracruz, FSC Celbi, Arauco Radiata, FSC BTMP Waggeryd and FSC CMPC Guaiba. Instead, the fillers amount was considered as the total amount of the different additives exploited to mix with the pulp. This simplification was made with the aim of reducing the variables number. Furthermore, the titanium dioxide was a preponderant element among the additives exploited in the different pulps considered. The additives considered for the process analysis were iron oxides, kaolin and inks. Other additives exploited but not considered for the analysis, as constant during the experimental investigation, were resins and lye.

After that, the refining process parameters were considered. The net refining power, which was the difference between load power and no-load power consumed by refiners, and the pulp flow were chosen, as their combination was an index of the refining intensity. Furthermore, the refining bar geometry of the conical refining disks, which was the shape of the bars providing for pulp refining and transport of fibre suspension, and their wear rate considered as the hours of operation were taken into account. In particular, the refiners exploited in this work were as follows: Parason SF (Parason, Aurangabad, India), Parason PA-2815SF (Parason, Aurangabad, India), Airaghi SF-2063019 (Officine Airaghi (S.R.L.), San Giovanni Lupatoto, Italy), Airaghi XSF-2063019 (Officine Airaghi (S.R.L.), San Giovanni Lupatoto, Italy) and Airaghi SF-20633019 (Officine Airaghi (S.R.L.), San Giovanni Lupatoto, Italy). In order to create a dataset suitable for the neural network training, five refining disk geometries exploited for the production were considered qualitatively. The last input parameter concerned the fibres morphology. Specifically, it is the mean length of the incoming fibres. The length of the fibres was chosen also as a parameter for the process control, since length modification is one of the main effects of the refining and a valuable index to evaluate the process effectiveness. The length measures were carried out by means of a fibres tester, Lorentzen & Wettre 912 plus (AB Lorentzen & Wettre, Kista, Sweden). It provides an automated fibres quality measurement of a pulp for fast classification and detection of fibres features, such as mean lengths, widths, areas, perimeters and shape factors, in primary and secondary fines amounts. In particular, the device calculates the weighted average fibre length, evaluated as the sum of individual fibre lengths squared divided by the sum of the individual fibre lengths. The boundary values of the variables considered in the dataset are reported in Table 1.

**Figure 2:**
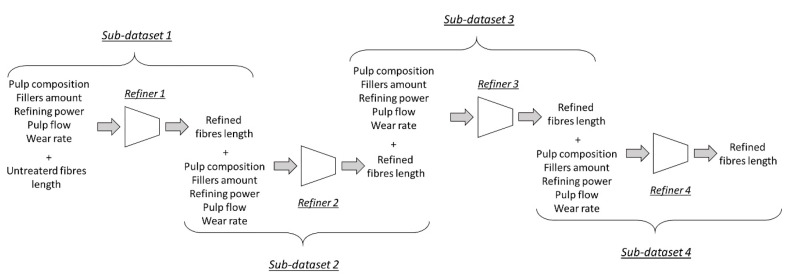
Subdataset creation scheme.

With the aim of acquiring examples to be exploited in the neural network training, a subdataset was acquired for each refiner involved in the cellulose refining, as represented in Figure 2.

Under the simplifying hypothesis that the refiners process the fibres in the same way, the experimental data extrapolated from each refiner were exploited to build a single dataset to be used in the ANN training. Additionally, the refiners were considered independent, so that, for a given stage, the effect of the previous refining step could be neglected. This is not rigorous, as there is a limit in the fibres lengthening during the refining and therefore in the energy that can be globally supplied to the fibres. The energy transferred to the fibres during the single refining step is low and does not affect the following refining stage. This allows for the development of a single neural network for the prediction of length modification after each step of the refining line considered in this study.

**Figure 3:**
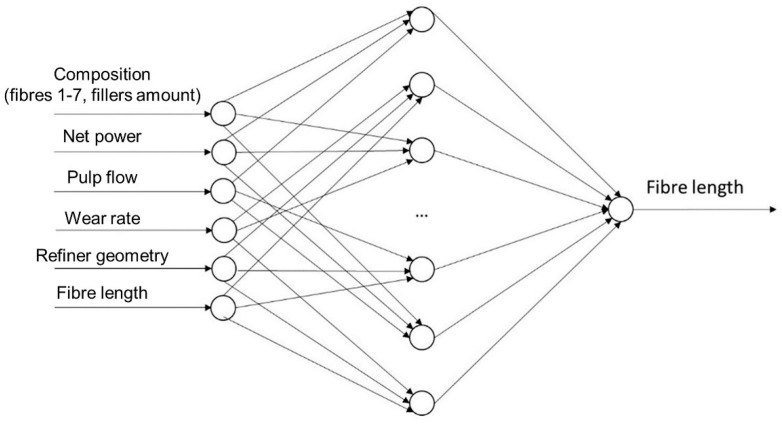
Schematic of the first artificial neural network (ANN1) structure.

The first ANN developed, named ANN1, is represented in Figure 3.

It was trained by means of the Broyden–Fletcher–Goldfarb–Shanno (BFGS) backpropagation algorithm exploiting 72 patters collected during the experimental phases previously described. The algorithm was chosen, because a quasi-Newtown method is one of the most suitable techniques for function fitting. Like any of Newton-like methods, BFGS uses the quadratic Taylor approximation of the objective function about a generic point x_n_, which can be written as Equation (1):

(1) f(xn+δx)=f(xn)+gnTδx+12δxTHnδx

where

(2) $$g_{n}=\nabla f\left(x_{n}\right)$$

(3) $$x_{n}=\nabla^{2}f\left(x_{n}\right)$$

The necessary condition for a minimum was described as:

(4) $$\nabla f\left(x_{n}\right)=g_{n}+H_{n}\delta x=0$$

(5) $$\delta x=-H_{n}^{-1}g_{n}$$

In an iterative process, the variables update was expressed as:

(6) $$\;x_{n+1}=x_{n}-H_{n}^{-1}g_{n}$$

In quasi-Newton methods, the idea is to use matrices that approximate, instead of computing, the Hessian matrix Hn for a reduced computational effort. Particularly, the BFGS method estimates the Hessian matrix with the following Equation (7):

(7) $$H_{n+1}=H_{n}-\;\frac{(H_{n}s_{n}){\left(s_{n}H_{n}\right)}^{T}}{H_{n}s_{n}s_{n}^{T}}+\frac{q_{n}q_{n}^{T}}{q_{n}^{T}s_{n}}$$

A connection between an input layer and a hidden layer was the tangent function, as it is most suitable in a hidden layer for ANNs aimed at function fitting. Although the pure linear transfer function is the most suitable for the connection of the hidden and output layers in order not to shorten a wide output range in a short interval, a logarithmic transfer function was applied. This was possible as the output values characterising the patterns in the dataset were compressed in a very short range. The numbers of neurons of the input and output layers were 13 and 1, respectively, representing the variables analysed. The hidden layer counted 25 neurons, which was the size giving the best prediction performance. It was the results of the test of several neural networks featuring the same architecture and different hidden sizes. The training of the network was performed exploiting 70% of the dataset for the training and the remaining 30% for the network validation. The stop criterion imposed was the maximum number of epochs equal to 2000 or the minimum square error equal than 1 × 10^−10^.

In order to enhance the neural network training, the dataset was treated by means of statistical tools. In fact, the process data exploited in the ANN1 training were handwritten in a specific paper register, as the IT (Information Technology) tools for data collection were missing in the paper mill object of the case study. This is a major source of uncertainty, which may cause a drop in the ANN performance. Particularly, a principal component analysis (PCA) was performed, excluding the data concerning the pulp fibres composition and the refiner geometry. It was mostly aimed at reducing the variance within the data introduced by inaccurate records of the raw materials quantities and the process parameters settings. Furthermore, the consequent input variables reduction aids the evaluation of the error surface, enhancing the ANN performance, reducing the ANN architecture’s size and the weights number. Additionally, the reduction of the variables number was approached, evaluating the correlation coefficients and performing a scatterplot analysis. At a later stage, a single-linkage hierarchical clustering (HC) was exploited, evaluating the Euclidean distances among the data for the outlier detection.

**Figure 4:**
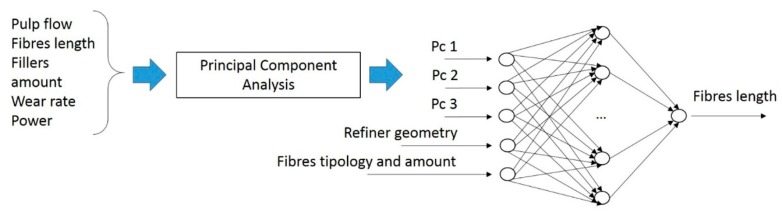
Schematic of the second artificial neural network (ANN2) structure.

The resulting modified dataset was applied for the training of the second artificial neural network (ANN2), of which the structure is represented in Figure 4.

It had an input layer featuring a reduced number of neurons with respect to the ANN1 implemented. In particular, the fibres typology and refiner geometry variables were kept unchanged. Instead, the pulp flow, fibres length, fillers amount, wear rate and refining power were elaborated by means of a PCA, which led to the definitions of three principal components. Therefore, the input layer counted 11 neurons, which referred to the input variable, while the output layer presented a single neuron in the representation of the fibres length. The hidden layer counted 7 neurons, which is the number of neurons to achieve the best performance. Again, the transfer functions between the layers were the tangent sigmoid and the logarithmic sigmoid transfer function for the output and hidden layers, respectively. As the size of this this ANN architecture was decreased with respect to the first attempt, the training algorithm was modified. In particular, the algorithm exploited was the the Levenberg–Marquardt algorithm, since it gives the best performances with small-size architecture when accurate training is required. The weight estimation was performed with the following equation, which produced a fester convergence of the training:

(8) $$x_{n+1}=x_{n}-{\left(H-\lambda diag\left[H\right]\right)}^{-1}g_{n}$$

For this reason, the minimum mean squared error and the epoch number were reduced with respect to the first ANN and set to 10^−10^ and 2000, respectively. The training was performed with 70% of the data left from the dataset cleaning, which was 52 patterns, while the test was conducted with the remaining 30% of the data. The split of the dataset was random.

While the performance of the networks was evaluated by means of the mean squared error, the error committed was evaluated as a ratio of the difference between the actual and predicted fibres lengths with an actual length as reported in the following Equation (9):

(9) $$E\%=\frac{L_{p}-L_{a}}{L_{a}}\times 100$$

where L_p_ is the predicted length and L_a_ is the actual length.

## 3. Results and Discussion

**Figure 5:**
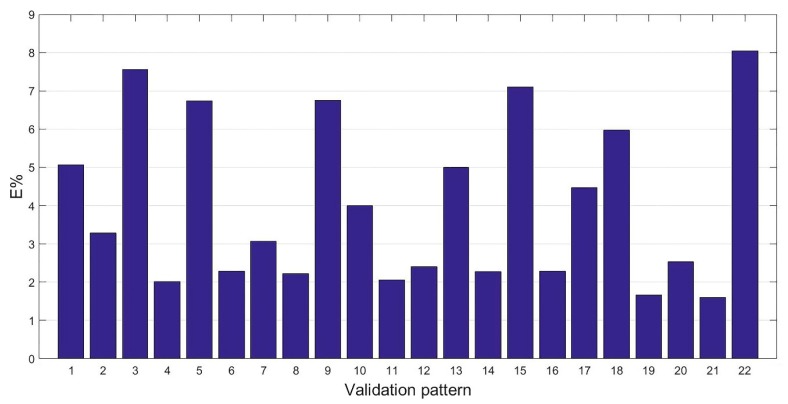
Validation results of the ANN1.

The results achieved with the ANN1 are reported in Figure 5, where E% committed for each pattern included in the validation set is reported.

**Figure 6:**
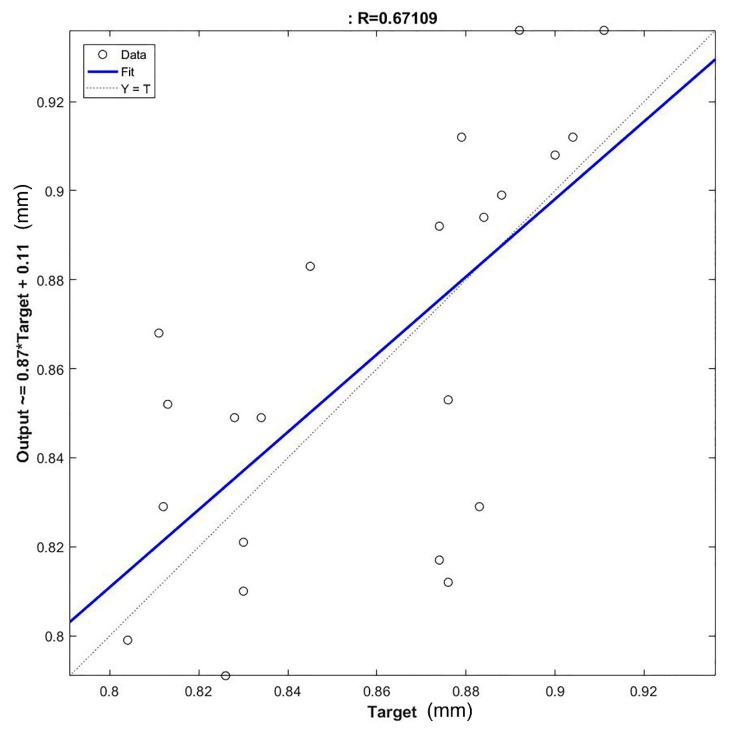
Regression plot of the ANN1 trained with the untreated data.

The mean percentage error committed by the network was 4.01%, which resulted in a difference between the actual and predicted lengths of about a hundredth of a millimetre the error committed by the network was not constant on the entire validation set. The validation featured a standard deviation of 2.16 with examples featuring errors up to 8%. Additionally, the regression plot of the model was performed and reported in Figure 6, which confirms the indication obtained by the E% analysis. Indeed, a rather low value of R^2^ = 0.67109 indicates the model does not represent a generalisation of the refining process under analysis.

These results may be caused by the presence of inaccurate patterns within the dataset due to the lack of information technologies. Indeed, the inaccurate data within the training set did not allow for the evaluation of a precise model. Therefore, the ANN created cannot replicate the actual manufacturing process and then offers an insufficient generalisation ability with a consequent high standard deviation of the mean percentage error. Furthermore, the outliers in the validation set may be responsible for those patterns featuring the highest error. Indeed, being outliers, those patterns cannot fit the model built in the training phase and, when the network is interrogated with the unknown data, produce elevated errors.

**Figure 7:**
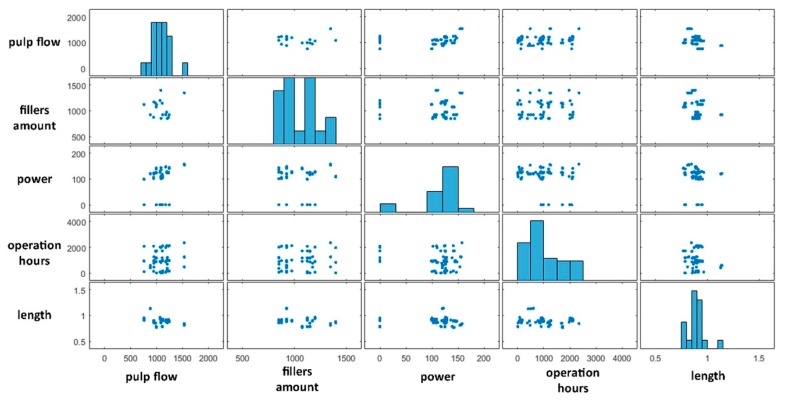
Scatter plot results.

As mentioned, both the problems were addressed with statistical analysis methods, HC and PCA. The statistical analysis comprised the evaluation of the correlation coefficient, reported in Table 2, and a scatterplot analysis, reported in Figure 7. The correlation coefficient showed that no linear correlation characterised the dataset. The same occurred with the scatterplot analysis, as no trend was highlighted, and the data were plotted as a cloud of points. Therefore, a nonlinear correlation was detected.

**Figure 8:**
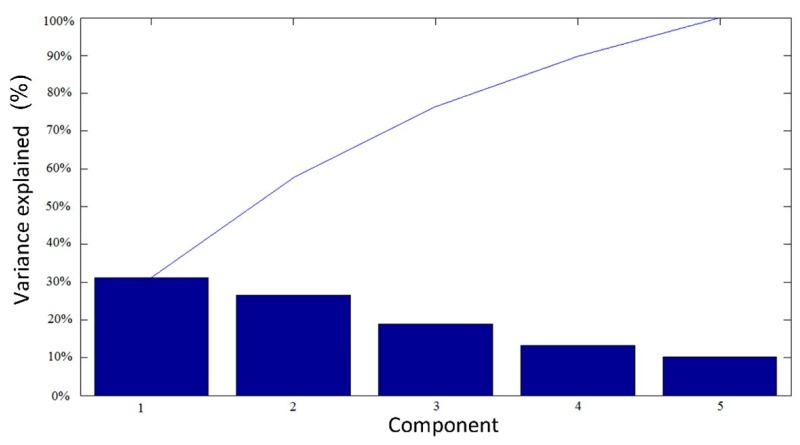
Principal component plotted as a function of the explained variance.

**Figure 9:**
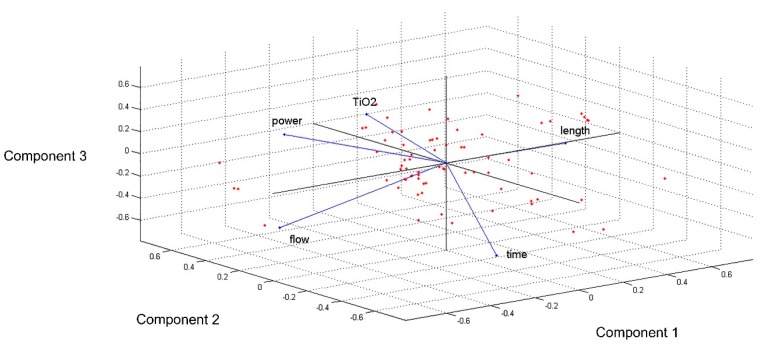
Patterns distribution in the principal components space.

Therefore, the dataset analysis proceeded with a PCA, of which the results are reported in Figure 8 and Figure 9.

Figure 8 highlights the results of the analysis, which was the variance explained by the principal components. Particularly, 82% the principal components can be compressed with the exploitation of three principal components; therefore, the variables included in the study were substituted by the first, second and third principal components. 

At a later stage, the distribution of the patterns among the principal component space was analysed.

**Figure 10:**
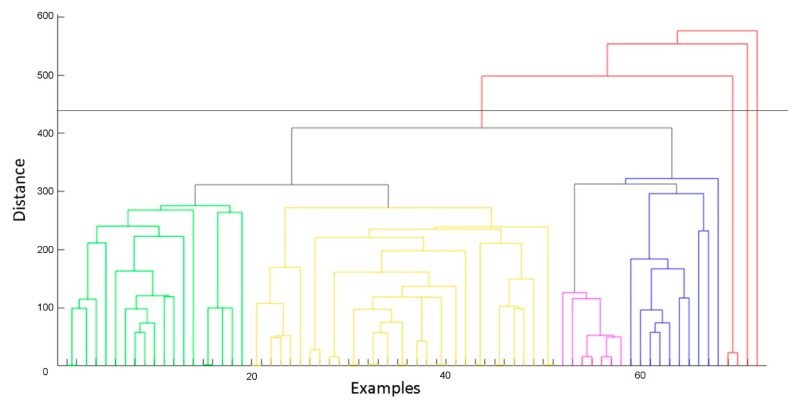
Hierarchical clustering results.

As reported in Figure 9, despite a central core of examples, aseveral patterns spreading to the boundary of the cloud of data analysed can be individuated. These patterns could be the outliers, and with the aim of detecting them, an HC was performed. The result is reported in Figure 10.

In the HC clustering reported while the horizontal axis represents the patterns or groups of patterns within the dataset, the vertical axis represents their geometrical distances in the multidimensional space of the variables. The shorter are the vertical segments connecting the patterns, and the closer and more similar are the examples analysed. It is evident how the dataset could be divided into four similar groups, where the distances among the examples are comparable. They can be associated to four similar kinds of paper produced in the paper mill during the study performed. Four subgroups of examples were characterised by an elevated distance from the other groups. These subgroups, which counted a total number of 19 patterns, were considered as outliers and not included in the dataset for the network training.

**Figure 11:**
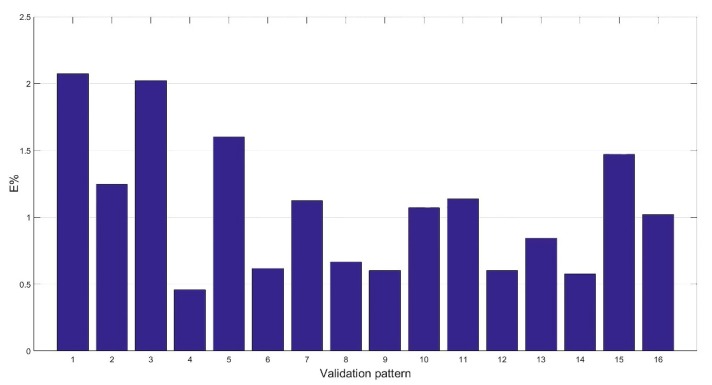
Validation results of the ANN2.

With the new dataset comprising the principal components instead of the actual variables and without the outliers detected, the training of ANN2 was performed. The validation results obtained are reported in Figure 11.

**Figure 12:**
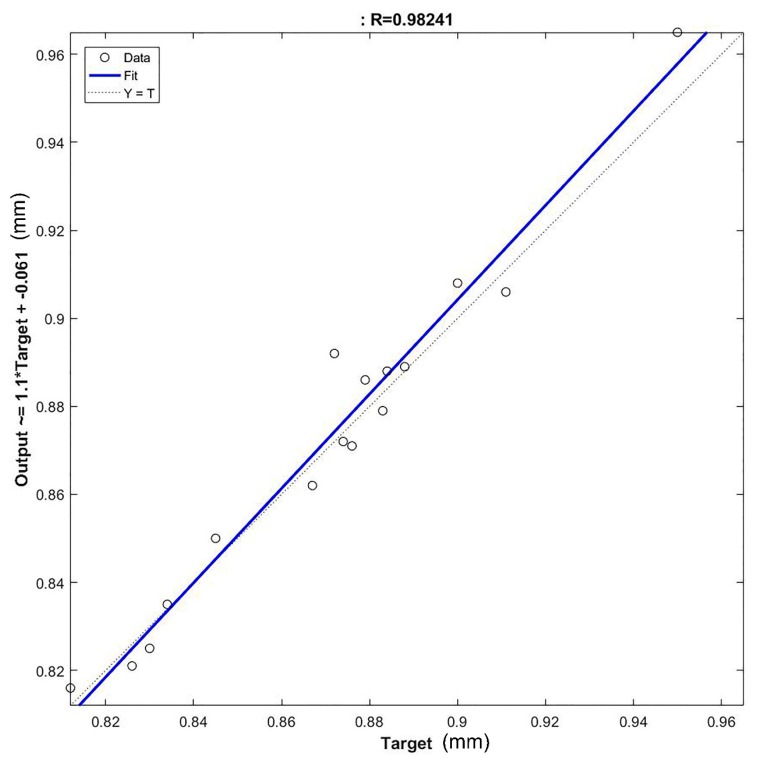
Regression plot of the ANN2 trained with the treated data.

It is evident the error committed was reduced. The network featured a mean error of 1.07% with a standard deviation of 0.5. Differently from the previous network implemented, the error was constant among the entire validation set, with a peak error comparable with the mean error of the previous network. It is inferable that the problems related to the dataset and the complexity of the system under examination were correctly addressed with the statistical analysis. The reduction of the input variables led to the definition of a simplified error surface, which allowed for the creation of a precise model capable to give a low mean percentage error with a reduced standard deviation. This is also visible considering the reduced number of neurons in the hidden layer necessary for the creation of an accurate model of the refining system. Additionally, the elimination of the outliers allowed obtaining a homogeneous dataset that led to the elimination of the highest error in the validation set. To validate the results obtained with the ANN2, a regression plot was performed to assess the similarity of the neural model built with the experimental data. It is reported in Figure 12.

An R^2^ value of 0.98 indicates the goodness of the model built, and therefore the model produced by ANN2 can be considered highly reliable. This is confirmed by the ANOVA, of which the results are reported in Table 3, which state the significance of the ANN model produced.

Later, the results of the network were analysed, considering not only the prediction accuracy but also the overall effect of the refining on the fibres. Particularly, the interest was to state the network ability in detecting cutting or stretching phenomena. For this reason, the difference between the initial and final lengths of the fibres was computed with the actual and predicted values to verify if they featured the same sign. In Table 4, the validation examples with a nonconcordant length difference are reported.

The example with a difference between the actual and predicted lengths concerned the process where the fibres were untreated. Indeed, all these examples feature a delta in the order of a thousandth of a millimetre. The accuracy of the network is in the order of a hundredth of a millimetre, and the variation of the length in a smaller range was not evaluated correctly. However, it is inferable that the networks have a great capacity in predicting the overall effect of refining on fibres. 

## 4. Conclusions

In the present paper, a case study with the development of a machine learning system for optimising a cellulose-refining system was proposed. An ANN was implemented and trained to model the fibres refining, a critical stage of paper manufacturing affecting both the paper mill effectiveness and the final product performance. On the basis of the process parameters exploited in the production process management, the ANN proposed allows for the prediction of the fibres length caused by the mechanical refining. The experimental dataset was treated by means of statistical tools; this led to the achievement of outstanding results in fibres length prediction. As this parameter is critical to achieve specific paper properties, its prediction allows for the optimisation of the additives amount and the process parameter to obtain specific properties of the final product.

## Figures and Tables

**Table 1:** Values of the variables analysed.

Variables	Min/Max
Fibres composition (1–7)	0/1
Fillers amount	850/1538 [kg]
Net power	0/158 [kw]
Pulp flow rate	760/1538 [L/min]
Wear rate	1/2352 [h]
Fibres length	0.768/1.140 [mm]

**Table 2:** Correlation coefficients.

	Pulp Flow	Fillers	Power	Time	Length
Pulp flow rate	1	0.061	0.272	0.081	−0.300
Fillers amount	0.061	1	0.029	0.080	−0.334
Power	0.272	0.029	1	−0.261	−0.010
Operation hours	0.081	0.080	−0.261	1	−0.148
Length	−0.300	−0.334	−0.010	−0.148	1

**Table 3:** ANOVA for the neural network model.

Source	DF	SS	MS	F-value	*P*-value
Model	1	0.021407	0.021407	387.57	1.3317 × 10^−11^
Residual	14	0.00077327	5.5234 × 10^−5^		
Total	15	0.02218	0.0014787		

**Table 4:** Validation examples with a nonconcordant length difference.

Input (mm)	Target (mm)	Prediction (mm)	Actual Delta (mm)	Predicted Delta (mm)
0.883	0.872	0.892	−0.009	+0.009
0.951	0.950	0.965	−0.001	+0.014
0.867	0.867	0.862	0	−0.005
